# Patent vitellointestinal duct with prolapse of inverted loop of small intestine: a case report

**DOI:** 10.1186/1752-1947-1-49

**Published:** 2007-07-14

**Authors:** Prashant N Mohite, Ashok M Bhatnagar, Virsing P Hathila, Jitendra H Mistry

**Affiliations:** 1 Department of Surgery, SSG Hospital & Medical College, Vadodara, Gujarat State, India; 2 Department of Surgery, New Civil Hospital, Surat, Gujarat State, India

## Abstract

A wide variety of anomalies may occur as a result of the vitellointestinal duct (VID) failing to obliterate completely. Most reports on symptomatic VID focus on Meckel's diverticulum, while other anomalies are given little attention. We report a case of a baby of five months who had an intestinal loop inverted through a patent VID. The inverted loop was reduced and ileostomy was done which was closed after 6 weeks.

## Background

### Anatomy

The midgut enlarges rapidly during the first 5 weeks of gestation and becomes too large for the abdominal cavity; subsequently, it is herniated into the umbilical cord. The apex of the herniated midgut is continuous with the vitellointestinal duct and the yolk sac. The axis of the herniated midgut is formed by the superior mesenteric artery. At approximately the 10th week of gestation, the midgut begins its return into the abdominal cavity. This return occurs by a highly complex developmental process, and as a result, numerous anomalies of the bowel may ensue. These include bowel atresias and stenoses, abnormalities of the vitellointestinal duct, failure of ceacal descent, malrotation, malfixation, reversed bowel rotation and exomphalos.

## Case presentation

5 months old male child came with unusual red colored 'Y' shaped loop emerging from the anterior abdominal wall with absent umbilicus. The parents reported that the baby had a small opening in the umbilicus since birth which discharged a sticky greenish material in very small quantity. The child was suffering from cough since 4 days leading to protrusion of the red colored mass from the umbilicus which was small initially but grew over these 4 days till it became carrot sized. Then a branch developed giving it a 'Y' shape.

**Figure 1:**
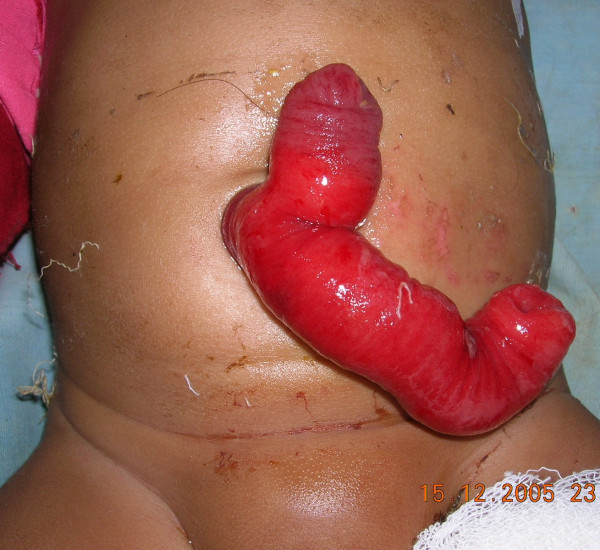
picture on presentation.

On careful examination we found that the loop was the 'y' shaped fork (See Figure 1). One of the tips of the mass was discharging sticky greenish fluids suggestive of intestinal juices. The stem of the 'Y' shaped loop was protruding from umbilicus and fixed to the anterior abdominal wall. The loop was irreducible and bled on touch suggestive of mucosal surface.

**Figure 2:**
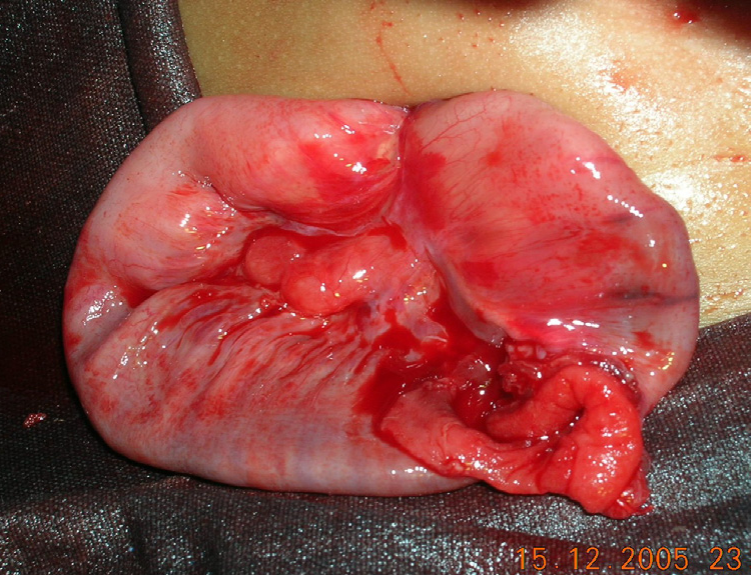
picture on reduction of prolapse.

**Figure 3:**
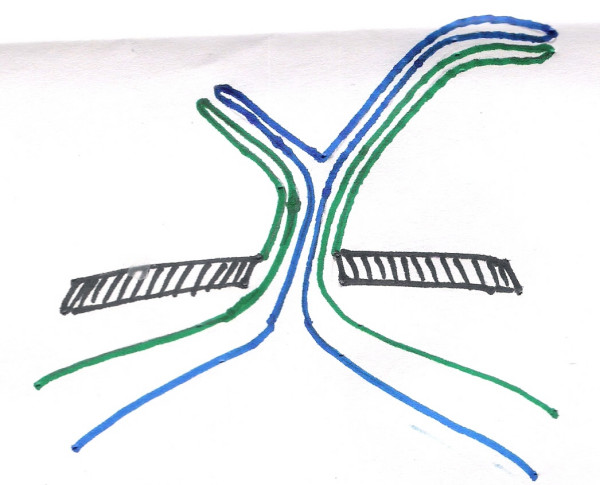
cross-sectional diagrammatic representation of the condition on presentation.

**Figure 4:**
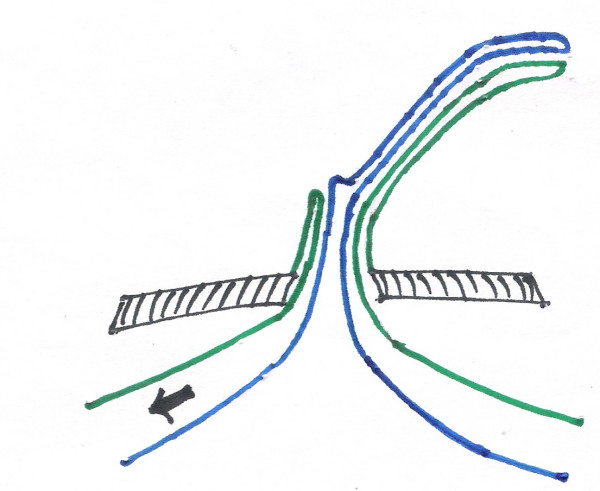
on reduction of the distal loop.

**Figure 5:**
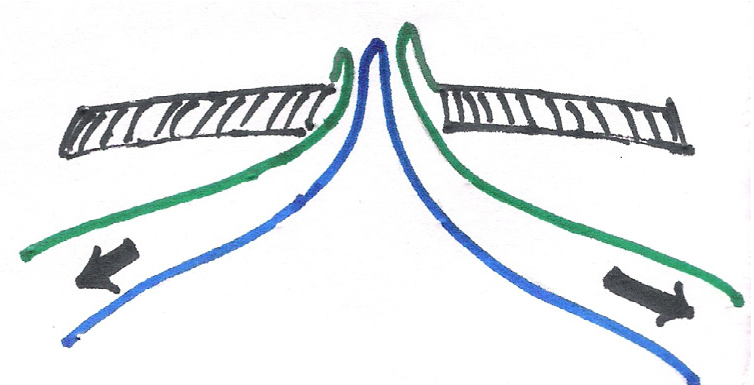
on reduction of proximal loop.

**Figure 6:**
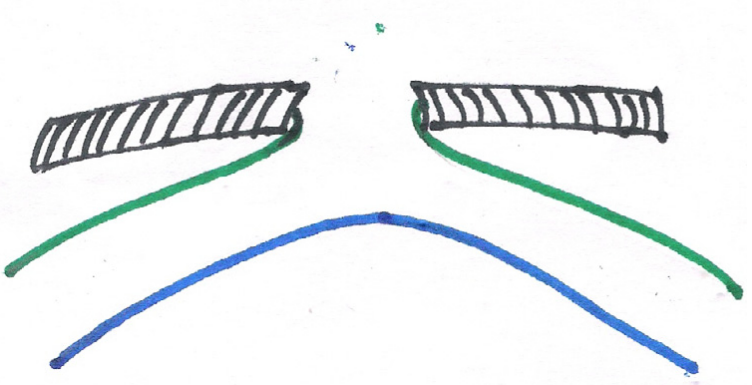
on complete reduction-with patent VID. Following color coding is used for all the figures 3 to 6. Black shaded area: abdominal wall. Blue: mesenteric border. Green: antimesenteric border

Ultrasound examination of the abdomen was found absolutely normal. Laparotomy was performed under general anaethesia. The baby was put through a laparotomy under general anesthesia. A small transverse incision of 4 centimeters was made just below the umbilicus. The outer surface of the emerging loop was firmly adherent to all the layers of the anterior abdominal wall. It was dissected from the abdominal wall layers with the fine scissors. Two different non-adherent loops of small intestine were found entering the emerging carrot like mass (See Figure 3). The distal loop entering into the umbilicus was slowly pulled inside the peritoneal cavity with extreme delicacy to find that the outer loop was diminishing in size and finally disappeared (See Figure 4). The procedure was applied for the proximal loop also (See Figure 5). After complete reduction (See Figure 6) a defect of 2 × 2 centimeters was found in the small intestine (See Figure 2) at the point of adherence with the abdominal wall suggesting its patency with external environment through the umbilicus. We diagnosed the case as patent vitellointestinal duct. Primary resection anastomosis was not possible because of the gross edema over the loop of intestine. The loop with the defect was brought out through another incision in the abdomen at right iliac fossa as loop ileostomy. The defect of Patent VID over the abdominal wall was primarily closed the umbilicus was reconstructed. Ileostomy was closed after 6 weeks. Patient condition was followed up for about 6 months with no reports of any complications.

## Discussion

Failure of obliteration of the embryonic vitellointestinal duct leads to various congenital anomalies like – Meckel's diverticulum, vitelline cord, enteric cyst, umbilical sinus, enteric fistula or hemorrhagic umbilical mass [1]. Patient may present the anomaly itself or due to complications secondary to the anomalies like intestinal obstruction due to volvulus, intussusception or adhesions [2,3]. Totally patent VID is a very rare anomaly and very few cases were reported in the literature. We found a single case report of ileal prolapse through the patent VID. It was reported in 1985 by Dr. Gvalani AK [4]. Patent vitellointestinal duct may present itself as continuous or intermittent discharge through the umbilicus. The defect which is wide enough or the predisposing conditions which increase intra abdominal pressure leads to partial or total prolapse of the intestine through the patent duct. The condition, if not managed promptly by surgical intervention, may lead to subacute or acute intestinal obstruction, strangulation and gangrene of the prolapsed intestinal loop. Primary closure of the VID following reduction of the prolapse may be possible if the patient arrives early without any gross edema over the intestinal loops. If the defect is large one can go for resection of the loop of intestine near the patent duct followed by primary anastomosis. If the patient arrives late with gross edema or the viability of the intestinal loops is in question then exteriorization of the suspected loop or loop ileostomy is advised.

## Conclusion

Patent VID with prolapsed ileal loop is a rare condition needs prompt diagnosis, surgical reduction and repair of the defect.

## Abbreviations

VID: vitellointestinal duct

## Competing interests

The author(s) declare that they have no competing interests.

## Authors' contributions

All the authors read and approved the manuscript.

PNM: Did the surgery, prepared the manuscript

AMB: Guided during surgery, helped in searching references.

VPH: Gave final touch to the manuscript

JHM: Assisted the surgery, assisted drafting the manuscript
